# The Alcohol Extract of* Coreopsis tinctoria* Nutt Ameliorates Diabetes and Diabetic Nephropathy in db/db Mice through miR-192/miR-200b and PTEN/AKT and ZEB2/ECM Pathways

**DOI:** 10.1155/2019/5280514

**Published:** 2019-03-28

**Authors:** Shunjie Yu, Haoran Zhao, Wenjing Yang, Ramila Amat, Jun Peng, Yike Li, Kai Deng, Xinmin Mao, Yi Jiao

**Affiliations:** ^1^Department of Biochemistry and Molecular Biology, School of Basic Medical Sciences, Xinjiang Medical University, Urumqi 830011, China; ^2^Traditional Chinese Medicinal College, Xinjiang Medical University, Urumqi 830011, China

## Abstract

The study aims to investigate the effects of the alcohol extract of* Coreopsis tinctoria *Nutt (AC) on diabetic nephropathy (DN) mice. A total of 30 db/db (DN) mice were divided into 3 groups, which were treated with AC (300 mg/kg/day), metformin (180 mg/kg/day), or saline by gavage for 10 weeks. Ten db/m mice treated with saline were used as normal control (NC group). Body weight (BW) and fasting blood glucose (FBG), HbA1c, 24 h urinary albumin excretion (UAE), and renal pathological fibrosis were analyzed. Expression of miR-192, miR-200b, and proteins in the PTEN/PI3K/AKT pathway was analyzed by qPCR or western blot. The DN mice had significantly higher BW, FBG, and 24 h UAE, as well as more severe pathological fibrosis when compared with NC. Treatment of AC could decrease BW, FBG, and 24 h UAE and alleviated kidney damage. Compared with the NC group, expressions of miR-192 and miR-200b were increased, whereas their target proteins (ZEB2 and PTEN) were reduced in the kidneys of DN mice, which further modulated the expression of their downstream proteins PI3K p85*α*, P-AKT, P-smad3, and COL4 *α*1; these proteins were increased in the kidneys of DN mice. In contrast, AC treatment reversed the expression changes of these proteins. These findings demonstrate that AC may protect the kidneys of DN mice by decreasing miR-192 and miR-200b, which could further regulate their target gene expression and modulate the activity of the PTEN/PI3K/AKT pathway to reduce the degree of renal fibrosis.

## 1. Introduction

Diabetes has become one of the major public health problems. In China, the prevalence of diabetes in adults reached 10.9% in 2013 [1–3]. Diabetic nephropathy (DN) is a common chronic microvascular complication of diabetes and is also an important cause of end-stage renal disease. Approximately 40% of patients with diabetes have DN [4, 5]. Glomerular structure hypertrophy, glomerular basement membrane thickening, mesangial cell proliferation, tubulointerstitial expansion, and the abnormal accumulation of extracellular matrix (ECM) components (such as collagen and fibronectin) are the main pathological features of DN [6]. The increase of collagen fibers is the main characteristic of renal fibrosis. During this process, the kidneys undergo excessive deposition of collagen, which causes gradual sclerosis and scarring of the renal parenchyma and eventually results in complete loss of kidney function [7].

The db/db mouse is a spontaneous diabetic model that exhibits many of the same characteristics as human diabetes. It has the typical clinical manifestations of diabetes such as hyperglycemia, hyperlipidemia, polyphagia, and polyuria. With age increasing, these mice can present early manifestations of DN, such as microalbuminuria. As the disease progresses, large amounts of proteinuria gradually develop, and the kidneys become diseased; eventually these effects develop into glomerulosclerosis and interstitial fibrosis [8, 9].

The occurrence and development of DN may be caused by oxidative stress, inflammatory reactions, glucose and lipid metabolism disorders, and other factors [10, 11]. These factors activate multiple signaling pathways in the kidney [12–14], which may contribute to the pathogenesis of DN. Among them, the PI3K/AKT signaling pathway is closely related to renal fibrosis [15]. Activation of the PI3K/AKT signaling pathway can phosphorylate smad3, promote the development of collagen fibers in mesangial cells, and increase the degree of renal fibrosis [16].

Moreover, some studies have found that the abnormal expression of microRNA (miRNA) is closely related to the occurrence and development of DN [17, 18]. One study found that the expression of miR-192 in the kidneys of DN mice can inhibit the autophagy process of mesangial cells and lead to glomerular hypertrophy [19]. In addition, increased expression of miR-200b activates the downstream PI3K/AKT signaling pathway, resulting in glomerular hypertrophy and collagen deposition [20].

Pharmacological studies [21–23] have shown that the alcohol extract of* Coreopsis tinctoria* Nutt has antihypertension, antihyperlipidemia, and hypoglycemic effects, as well as anti-inflammatory and antifibrosis effects in DN [24]. However, the underlying mechanism is unclear.

In this study, the role and mechanism of AC in DN was investigated. The db/db mice were used as research subjects. After daily administration of AC for 10 weeks, the effects of AC on body weight (BW) and biochemical indexes were observed to monitor the therapeutic effect of AC on DN. Furthermore, expression of miR-192, miR-200b, and their target genes that belong to the phosphatase and tensin homolog deleted on the chromosome ten (PTEN)/phosphatidylinositol 3-kinase (PI3K)/protein kinase B (AKT) signaling pathway in the kidney tissues of DN mice was analyzed to investigate the possible mechanism underlying the effect of AC.

## 2. Materials and Methods

### 2.1. Preparation of AC

Dried flowers of* Coreopsis tinctoria* Nutt (the species was identified by Professor Junping Hu, College of Pharmacy, Xinjiang Medical University) were crushed and placed in 55% ethanol for reflux extraction. The extracted liquid was filtered and merged. The filtrate was concentrated in a concentration tank under reduced pressure and then concentrated by a multistage flash evaporator (DC-NSG; Jinding Technology, Henan, China). The concentrate was evaporated to approximately 600 ml in a rotary evaporator (R-210; Buchi, Essen, Germany) and then vacuum-dried to obtain AC. AC was passed through HPD-100 macroporous resin and then eluted with 75% ethanol. The ethanol elute was vacuum-dried to obtain the purified AC. The purified AC was accurately weighed at 0.5 g. 10 ml 60% ethanol was added to dissolve the purified AC. The solution was passed through a 0.22 *μ*m microporous membrane, and the resulting filtrate was used as the AC solution (0.05 g/ml).

### 2.2. Analysis of AC by High Performance Liquid Chromatography (HPLC)

The compounds in AC were analyzed by HPLC (Shimadzu, Japan). AC was separated by a Shim-pack VP-ODS column (150 mm × 4.6 mm, 5 *μ*m). The mobile phase contained 0.5% solution A (formic acid) and solution B (acetonitrile), with a detection wavelength of 280 nm, column temperature of 35°C, and flow rate of 1.0 mL/min. The elution program was as follows: 0 ~ 60 min, 95 ~ 80% solution A and 5 ~ 20% solution B; 60 ~ 75 min, 80 ~ 10% solution A and 20 ~ 90% solution B; 75 ~ 80 min, 10 ~ 95% solution A and 90~5% solution B. According to the Chinese Pharmacopoeia on HPLC (General Rule 0512), the chlorogenic acid concentration (C) was plotted on the abscissa and the peak area (A) was plotted on the ordinate to obtain the standard curve. The regression equation was as follows: A = 13038C - 19022, r = 0.9991. Then, 10 *μ*L of the chlorogenic acid reference solution (400 *μ*g/mL) or the AC solution was added to a liquid chromatograph, respectively. The peak area of chlorogenic acid was used as a control to calculate the content of flavanomarein, marein, and 3,5-dicaffeoylquinic acid in AC.

### 2.3. Animals

Ten db/m mice (4 weeks old) and 30 db/db mice (4 weeks old) were obtained from Caveven's Laboratory Animal Co., Ltd., (Changzhou, China). The animals were kept in a specific pathogen-free environment in a 12 h light/dark cycle at 21±2°C and 45±5% humidity for 1 week of acclimatization. All mice were allowed free access to normal food and water. The study was approved by the Institutional Animal Care and Use Committee of the First Affiliated Hospital of Xinjiang Medical University (Approval No. IACUC-20140304-158).

### 2.4. Animal Treatment and Grouping

The db/m mice (n=10) were used as normal control (NC). The db/db mice were randomly divided into three groups, the DN group, DN+M group, and DN+AC group, with 10 mice in each group. Mice in the NC group and DN group were given saline by gavage each day for 10 weeks. Mice in the DN+M group received 180 mg/kg metformin (Squibb Pharmaceutical Co., Ltd., Shanghai, China) by gavage each day for 10 weeks. 300 mg/kg purified AC was administrated to the DN+AC group by gavage each day for 10 weeks.

### 2.5. Specimen Collection

The BW of all mice was obtained every two weeks. Caudal blood was collected from all mice every two weeks, and fasting blood glucose (FBG) levels were measured. Once every two weeks, urine was collected for 24 h, and urine volume was recorded. After 10 weeks of gavage, the mice were fasted for 8 h and then blood was collected from the eyeballs after anesthesia by intraperitoneal injection of 2% pentobarbital sodium (40 mg/kg). Blood samples were used for detection of HbA1c by HPLC. After that mice were sacrificed by cervical dislocation. The kidneys were dissected. The right kidneys were kept immediately in liquid nitrogen for subsequent extraction of total RNA and protein. The left kidneys were weighed to calculate kidney/weight ratio and then fixed in 10% formaldehyde.

### 2.6. Glucose Oxidase Assay

Fasting blood glucose was measured by a glucose assay kit (the glucose oxidase method) (BioVision, Mountain View, CA, USA). Briefly, the blank wells with 20 *μ*L 0.1% NaF, standard wells with 0.28 mmol/L, 0.56 mmol/L, and 1.11 mmol/L of glucose standard, and wells with plasma were set up on a 96-well plate. The volume of each well was 20 *μ*L. Then, 180 *μ*L of glucose oxidase was added to each well and incubated at 37°C for 45 min at 200 rpm. The optical density of each well was measured at a wavelength of 505 nm with a microplate reader (xMark, Bio-Rad, Hercules, CA, USA). A standard curve was generated and the fasting blood glucose concentration was calculated.

### 2.7. ELISA

The content of urinary albumin was determined by a mouse urinary albumin ELISA kit (Chenglin Biological Technology Co., Ltd., Beijing, China). Briefly, blank wells, standard wells (360 *μ*g/L, 240 *μ*g/L, 120 *μ*g/L, 60 *μ*g/L, and 30 *μ*g/L), and sample wells were set on the ELISA plate. Then, 10 *μ*L of mouse urine was added to each sample well, and the plate was incubated at 37°C for 30 min. After washing, 50 *μ*L reagent A and 50 *μ*L reagent B were added to each well and incubated at 37°C for 15 min in the dark. The reaction was stopped by adding a 50 *μ*L stop solution. The absorbance of each well was measured by a microplate reader (Bio-Rad) at 450 nm wavelength.

### 2.8. H&E and Masson's Staining

The left kidney tissues were fixed in a 10% formaldehyde solution for 24 h, and then, tissue sections were prepared and subjected to hematoxylin and eosin (H&E) and Masson's staining as follows. First, the tissue was dehydrated with graded ethanol and transparentized with xylene. Then, the tissue was embedded in paraffin and cut into 3~4 *μ*m sections. The tissue sections were stained with H&E and Masson's according to routine procedure. The morphological changes of the kidneys were observed under a light microscope (SZX7-1093; Olympus, Japan). Histological evaluation was performed in a blinded manner. The mesangial expansion index from 30 glomeruli of each mouse was graded in four levels from 0 to 3 [24]: grade 0, normal glomerulus; grade 1, matrix expansion occurring in up to 50% of a glomerulus; grade 2, 50%–75% expansion; and grade 3, 75%–100% expansion. For Masson's staining, four levels of fibrosis from 30 glomeruli of each mouse were scored by measuring the percentage area of blue-purple staining as follows: grade 0, absent or < 25%; grade 1, 25%–50%; grade 2, 50%–75%; and grade 3, 75%–100%, and the mean score of each mouse was calculated and compared. A total of 5 mice in each group were analyzed.

### 2.9. Real-Time Quantitative PCR (qPCR)

Total RNAs of the renal tissues were extracted with the miRNA extraction kit (Sangon Biotech Co., Ltd., Shanghai, China). Reverse transcription was performed using a miRNA first-strand cDNA (synthesis tail method) kit (Sangon Biotech Co., Ltd., Shanghai, China). Specific primers for U6, miR-192, and miR-200b were designed by Qiagen (Valencia, CA, USA), and the primer sequences (5′→3′) were AACGCTTCACGAATTTGCGT, CTGACCTATGAATTGACAGCC, and TAATACTGCCTGGTAATGATGA, respectively. qPCR was carried out in 20 *μ*L final volumes with 2 *μ*L cDNA, 3 *μ*L RNase-free H_2_O, 10 *μ*L 2× miRNA qPCR Master Mix (Sangon, Shanghai, China), 2 *μ*L universal PCR primer, 1 *μ*L ROX Reference Dye (Sangon, Shanghai, China), and 2 *μ*L specific primers. qPCR was performed with a real-time PCR thermocycler (Applied Biosystems, Foster City, CA, USA) under the following conditions: predenaturation at 95°C for 5 s and then 40 cycles of denaturation at 95°C for 30 s and annealing/extension at 60°C for 30 s. miR-192 and miR-200b relative expression levels were calculated using the 2^−ΔΔCt^ method and normalized to the expression of U6.

### 2.10. Western Blotting

The right kidneys were lysed with the RIPA lysis buffer (Thermo Fisher Scientific, Waltham, MA, USA) containing a protease and phosphatase inhibitor cocktail (Abcam, Cambridge, MA, USA). The supernatant was collected by centrifugation at 12000 rpm for 10 min, and the total protein concentration was measured by the BCA method. Proteins were separated by electrophoresis and transferred onto PVDF membranes (Millipore, USA). After blocking in 5% nonfat milk at room temperature for 1 h, the membranes were incubated at 4°C overnight with diluted primary antibodies to ZEB2, PTEN, PI3K p85*α*, AKT, P-smad3, smad3, and COL4 *α*1 (all with 1:1000 dilution; Abcam, Cambridge, MA, USA), *β*-actin (1:2000; ZSGB-BIO, Beijing, China), and P-AKT (1:5000; Abcam, USA). After rinsing, the membranes were incubated with an HRP-labeled anti-mouse or anti-rabbit secondary antibody (1:5000; ZSGB-BIO, Beijing, China) for 1 h at room temperature. After rinsing, the membranes were developed with enhanced chemiluminescence (Thermo Fisher Scientific, USA). Images were acquired by an image acquisition system (Gel Doc XR+; Bio-Rad, USA), and the grey values were determined by ImageJ software.

### 2.11. Statistical Analysis

SPSS 19.0 statistical software (Chicago, IL, USA) was used for analysis. The data were expressed as the means ± standard error. The differences among multiple groups were analyzed by one-way analysis of variance.* P*<0.05 was considered statistically significant.

## 3. Results

### 3.1. HPLC Analysis of AC

**Figure 1:**
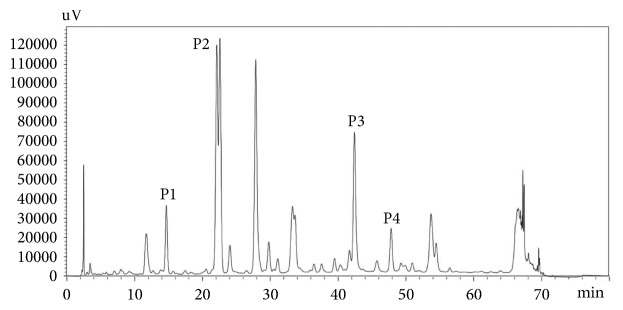
*HPLC analysis of AC*. HPLC analysis of AC at 280 nm wavelength. The four main compounds were chlorogenic acid (P1), flavanomarein (P2), marein (P3), and 3,5-dicaffeoylquinic acid (P4).

AC contains amino acids, polysaccharides, volatile oils, flavonoids, and organic phenolic acids. Among them, flavonoids (flavanomarein, marein) and phenolic acids (chlorogenic acid, 3,5-dicaffeoylquinic acid) are the main active components of AC and are closely related to the pharmacological action of* Coreopsis tinctoria* Nutt [25–27]. By HPLC analysis, we found that chlorogenic acid, flavanomarein, marein, and 3,5-dicaffeoylquinic acid were the main flavonoids and phenolic compounds of AC. The content of these bioactive components in AC was determined by a quantitative assay of multiple components with chlorogenic acid as a single marker, as shown in Figure 1 and Table 1.

### 3.2. Change in BW and FBG after Treatment with AC

To observe the effect of AC on the weight of the DN mice, BW was monitored. We found that mice from the DN, DN+M, and DN+AC groups were significantly heavier than those of the NC group from 0-10 weeks (*P*<0.05) (Table 2). Compared with that of the DN group, the BW of mice in the DN+M group was lower at 8 and 10 weeks (*P*<0.05), and the BW of the DN+AC group mice was also significantly lower at 10 weeks (*P*<0.05) (Table 2). To observe the effect of AC on the FBG levels of the DN mice, we tested FBG every 2 weeks and found that the FBG in all the DN groups increased significantly compared with that of the NC group from 0 to 10 weeks (*P*<0.05). Compared with that of the DN group, the mice in the DN+M group had lower FBG (*P*<0.05) at 8 weeks, whereas the FBG of the mice in the DN+AC group was lower at 4, 6, 8, and 10 weeks (*P*<0.05) (Table 2). These results suggest that AC can reduce the elevated BW and FBG levels in DN mice.

### 3.3. Effects of AC on HbA1c and Kidney/Weight Ratio (K/W)

To observe the effects of AC on blood glucose control and kidney damage in DN mice, we measured the level of HbA1c and the K/W value at 10 weeks. Compared with the NC group, the HbA1c level and K/W of the mice in the DN group were significantly increased (*P*<0.05) (Table 3). The DN+M group had lower K/W (*P*<0.05) than the DN group, while the DN+AC group had both lower HbA1c and lower K/W (*P*<0.05) (Table 3). These results suggest that AC can reduce HbA1c level and K/W in DN mice.

### 3.4. Change in 24 h Urine Volume (24 h UV) and 24 h Urinary Albumin Excretion (24 h UAE) after Treatment with AC

To observe the effects of AC on 24 h UV and 24 h UAE in DN mice, we collected the 24 h urine of mice and detected the 24 h UAE every 2 weeks. Compared with those of the NC group, the 24 h UV and 24 h UAE of mice in each DN group were significantly higher at 2, 4, 6, and 8 weeks (*P*<0.05) (Table 4). Compared with that of the DN group, the 24 h UV in the DN+M group decreased at 4 and 8 weeks, and the 24 h UAE in the DN+M group decreased at 8 weeks (*P*<0.05) (Table 4). At both 6 and 8 weeks, the 24 h UV and UAE decreased in the DN+AC group (*P*<0.05) (Table 4). These results suggest that AC can reduce 24 h UV and 24 h UAE in DN mice.

### 3.5. Effect of AC on Renal Histology

**Figure 2:**
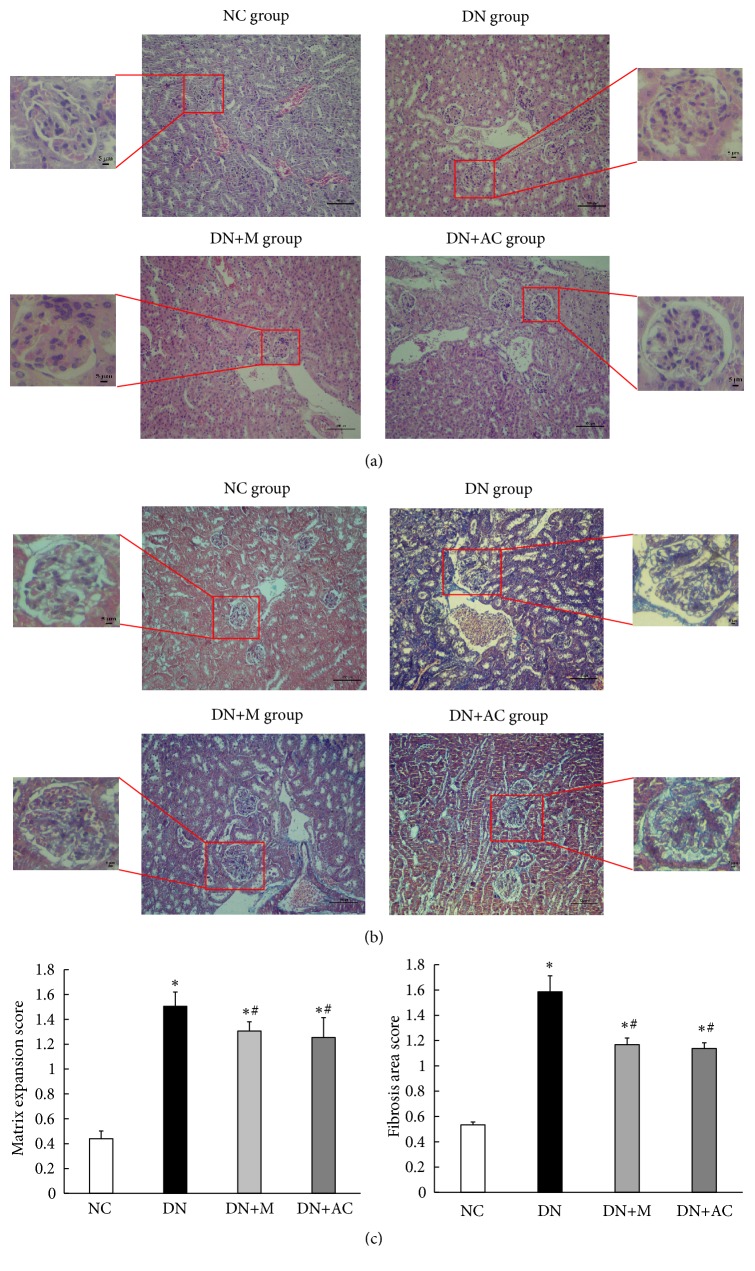
*Renal tissue morphology in four groups of mice*. Mice were divided into the NC group, DN group, DN+M group, and DN+AC group. (a) H&E staining of renal tissue morphology in four groups of mice. (b) Masson's staining of renal tissue morphology in four groups of mice. Large figures (200 x), scale bar, 100 *μ*m, and small figures (400 x), scale bar, 5 *μ*m. (c) Matrix expansion and area of fibrosis were semiquantitatively analyzed from 30 glomeruli per mouse with 5 mice in each group. *∗*:* P*<0.05 compared with the NC group; #:* P*<0.05 compared with the DN group, one-way analysis of variance.

At the end of treatment, renal tissues were examined by H&E staining and Masson's staining. As shown in Figure 2(a), the glomeruli of the mice in the NC group had a clear structure, normal volume, normal mesangial matrix, and clear and patent tubular lumen. In the DN group, the glomerular volume of most mice was obviously increased, the mesangium was enlarged, the basement membrane was thickened, and there were vacuole-like changes in the renal tubules. In the DN+M and DN+AC groups, the pathological changes of DN were alleviated (*P*<0.05) (Figure 2(c)). The glomerular volume was smaller than that of mice in the DN group; in addition, the mesangium showed mild widening, the basement membrane was mildly thickened, and the tubular vacuole-like changes were reduced. By Masson's staining, no obvious blue-purple collagen fibers in the glomerular, tubular, or renal interstitials were observed in the NC group mice. In the DN group, the staining of the blue-purple collagen fibers was obvious, and the deposition of renal interstitial collagen fibers was obvious. The blue-purple collagen fibers in the glomerular, tubular, and renal interstitials of the DN+M and DN+AC groups were fewer than those in the DN group (*P*<0.05) (Figures 2(b) and 2(c)). These results suggest that AC and metformin can alleviate DN mouse kidney mesangial widening, basement membrane thickening, and tubular vacuolar changes, as well as the deposition of collagen fibers, therefore playing a protective role in DN mouse kidneys.

### 3.6. Effects of AC on the Expression of miR-192 and miR-200b in Mouse Kidneys

**Figure 3:**
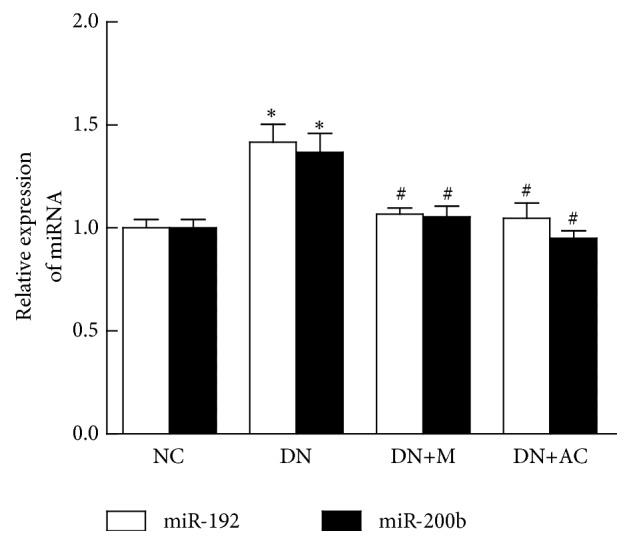
*Effects of AC on the expression of miR-192 and miR-200b in mouse kidneys.* Expression of miR-192 and miR-200b in mouse kidneys was detected by qPCR. U6 snRNA was used as internal control. *∗*:* P*<0.05 compared with the NC group; #:* P*<0.05 compared with the DN group, one-way analysis of variance.

To investigate the expression of miR-192 and miR-200b in the kidneys of DN mice and to observe the effects of AC on the expression of these miRNAs, qPCR was performed. The expression of miR-192 and miR-200b was significantly increased in the DN group compared with NC (*P*<0.05); however, treatment with metformin or AC significantly decreased the expression of these miRNAs (*P*<0.05) (Figure 3). These results suggest that AC can reduce the overexpression of miR-192 and miR-200b in the kidneys of DN mice.

### 3.7. Effects of AC on Target Gene Expression and the PI3K/AKT Signaling Pathway

**Figure 4:**
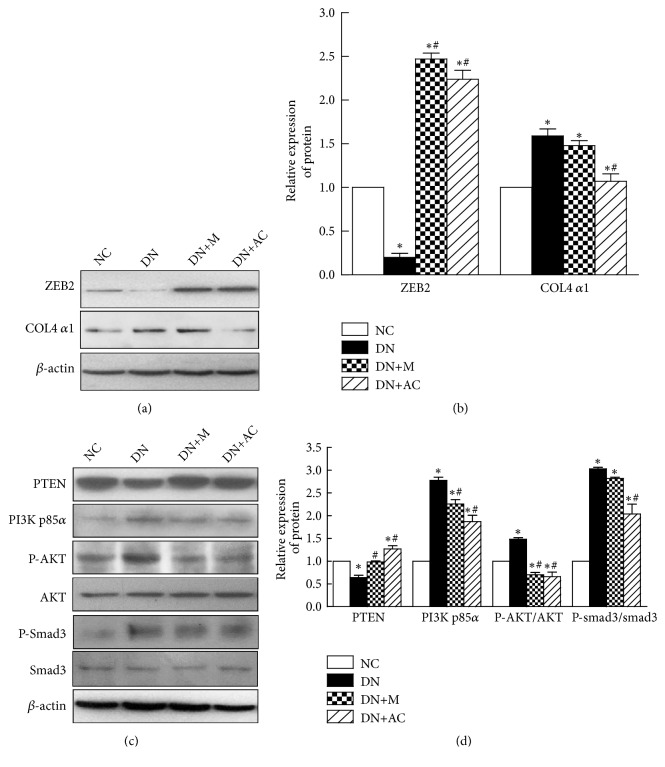
*Effects of AC on target gene expression and the PI3K/AKT signaling pathway*. Western blotting was used to detect protein expression. (a) Effect of AC on the protein expression levels of ZEB2 and COL4 *α*1 in mouse kidneys. (b) Grey level analysis of ZEB2 and COL4 *α*1 bands. (c) Effect of AC on the protein expression levels of PTEN, PI3K p85*α*, P-AKT/AKT, and P-smad3/smad3 in mouse kidneys. (d) Grey level analysis of PTEN, PI3K p85*α*, P-AKT/AKT, and P-smad3/smad3 bands. *∗*:* P*<0.05 compared with the NC group; #:* P*<0.05 compared with the DN group, one-way analysis of variance.

To explore the mechanisms underlying the protective effects of AC on the kidneys of DN mice, the expression of the miR-192 target gene ZEB2 and the miR-200b target gene PTEN was determined by western blotting. Compared with the NC group, the expression of ZEB2 and PTEN protein in the kidneys of the DN group was significantly decreased, which could be further rescued by the addition of metformin or AC (Figures 4(a)-4(d)). Next we examined the expression of proteins in the PI3K/AKT signaling pathway, since this pathway is thought to play an important role in renal fibrosis. Compared with the NC group, the expression of PI3K p85*α*, P-AKT (Ser473), P-smad3 (Ser425), and COL4 *α*1 in DN mouse kidneys was significantly increased (*P*<0.05). In addition, the expression of PI3K p85*α* and P-AKT (Ser473) in the DN+M group was decreased significantly, and the expression of PI3K p85*α*, P-AKT (Ser473), P-smad3 (Ser425), and COL4 *α*1 in the DN+AC group was decreased significantly (*P*<0.05) when compared with the DN group (Figures 4(a)-4(d)). These results suggest that the anti-renal-fibrosis activity of AC may be related to increased expression of ZEB2 and inhibited activation of the PTEN/PI3K/AKT signaling pathway.

## 4. Discussion

DN is a chronic complication secondary to diabetes. It is reported that DN has a 5-year survival rate of only 20% in patients with kidney failure [28]. Although understanding of the molecular mechanism of DN has progressed in recent years [29, 30], therapies to prevent or reverse the progression of DN are still lacking.

*Coreopsis tinctoria* Nutt, also known as Kunlun snow chrysanthemum, is a natural plant in Xinjiang [31]. Studies have shown that AC has a variety of biological and pharmacological activities [21–23]. Our previous study showed that both AC and the main flavonoid, marein, suppressed rat glomerular mesangial cell (HBZY-1) hyperplasia and significantly attenuated the expression of high glucose-disrupted fibrotic and inflammatory proteins in HBZY-1 cells. In addition, we also found that AC and marein may decrease renal inflammation and fibrosis via transforming growth factor-*β*1 (TGF-*β*1)/Smads, AMP-activated kinase protein (AMPK) signaling [32]. However, the effect of AC treatment on DN was not completely elucidated and needs more exploration. Therefore, in this study, we showed the protective effect of AC on DN renal fibrosis and found the possible underlying mechanisms.

We used db/db mice as the research subjects and successfully established the DN model. Here we observed that, after AC intervention, the FBG and HbA1c level, 24 h UV, 24 h UAE, and K/W were decreased in DN mice, and the structural abnormalities and fibrosis in the kidneys were significantly improved, indicating that AC has a protective effect on the kidneys of DN mice.

Many studies have shown that miRNAs play an important role in the pathogenesis of DN, and the abnormal expression of some miRNAs is closely related to the occurrence and development of DN [33–35]. The expression of miR-192 and miR-200b is known to be increased in the kidneys of DN mice [17]. ZEB2 can bind to the miR-200b and collagen gene promoter E-boxes and inhibit the transcription of the miR-200b and collagen genes [36–38]. Reduction of ZEB2 by miR-192 results in increased expression of miR-200b and the collagen gene [17]. Consistent with the results of these studies, we found a significant increase in the expression of miR-192, miR-200b, and COL4 *α*1 and a decrease in ZEB2 expression in the kidneys of DN mice. In contrast, AC intervention reduced miR-192 and miR-200b expression, increased ZEB2 expression, and decreased COL4 *α*1 expression. These results suggest that the protective effect of AC on DN may be achieved by regulating the expression level of miRNAs.

Studies have shown that PTEN is the target gene of miR-200b by luciferase reporter assay [39]. PTEN, or phosphatidylinositol-3,4,5-triphosphate (PIP3) phosphatase, can dephosphorylate PIP3 to phosphatidylinositol-4,5-bisphosphate (PIP2), thereby antagonizing the action of PI3K, to achieve negative regulation of the PI3K/AKT pathway [40]. Many studies have shown that PI3K/AKT signaling pathway activation is closely related to ECM increase in the kidney [41, 42]. The decreased expression of PTEN and the activation of the PI3K/AKT pathway in DN rat kidneys may result in increased 24 h UAE, renal fibrosis, and mesangial cell hypertrophy in rats [43, 44]. After AKT is activated by phosphorylation, the 3'C serine site of smad3 can be phosphorylated [16]. Phosphorylated smad3 enters the nucleus and binds to the promoter of the collagen gene, increasing the synthesis of collagen fibers, which results in increased ECM in the kidneys, a widened mesangium, and increased basement membrane thickness [45].

Our results showed decreased expression of PTEN, increased expression of PI3K p85*α*, P-AKT (Ser473), P-smad3 (Ser425), and COL4 *α*1, with an expanded mesangial matrix, a thickened basement membrane, and severe collagen deposition in DN mice when compared with normal mice. These results are consistent with previous findings [15, 16], indicating that activation of the PI3K/AKT pathway is closely related to renal morphological changes and fibrosis. In contrast, AC treatment significantly increased the expression of PTEN in DN mice. The expression of PI3K p85*α*, P-AKT (Ser473), P-smad3 (Ser425), and COL4 *α*1 was decreased, and the renal pathological changes were alleviated upon AC treatment. These data collectively indicated that AC alleviates the production of collagen and the degree of fibrosis possibly through the PI3K/AKT pathway. Although we previously showed that marein may be one of the bioactive compounds in AC [32], the activity of other components in AC still needs to be determined in order to identify which component is the most effective one. We will continue to study other possible mechanisms by which AC alleviates DN and whether the components of AC have a synergistic effect on mitigating DN.

## 5. Conclusions

AC can reduce the BW, FBG, HbA1c, and 24 h UAE of DN mice and play a protective role in DN mouse kidneys. This protective effect may be achieved by the indirect modulation of the activity of the PTEN/PI3K/AKT pathway through miR-192 and its target gene ZEB2. These results may provide the theoretical basis and treatment scheme for the clinical treatment of DN. However, we will further investigate the specific mechanism by which AC inhibits miR-192 expression in the future.

## Figures and Tables

**Table 1:** Contents of the main flavonoids and phenolic acids in AC (mg/g).

Peak no.	Compounds	Content
P1	Chlorogenic acid	26.27
P2	Flavanomarein	138.5
P3	Marein	268.56
P4	3,5-Dicaffeoylquinic acid	16.39

**Table 2:** Effect of AC on body weight and fasting blood glucose in each group ($\overset{-}{x}$±SE, g, mmol/L).

Weeks	NC		DN		DN+M		DN+AC	
	BW (g)	FBG (mM)	BW (g)	FBG (mM)	BW (g)	FBG (mM)	BW (g)	FBG (mM)
0 W	16.87±0.46	4.26±0.22	30.46±1.63*∗*	9.34±0.98*∗*	30.61±0.83*∗*	8.39±0.33*∗*	30.64±1.42*∗*	8.18±0.78*∗*
2 W	20.89±0.39	4.85±0.13	35.33±2.24*∗*	12.77±1.15*∗*	33.02±1.06*∗*	10.91±0.99*∗*	33.53±1.60*∗*	10.04±1.04*∗*
4 W	21.28±0.31	5.24±0.42	38.56±2.02*∗*	14.02±1.94*∗*	34.67±1.52*∗*	12.15±1.48*∗*	35.35±1.66*∗*	9.92±1.15*∗*#
6 W	23.45±0.45	5.34±0.15	41.50±2.24*∗*	15.26±1.78*∗*	37.39±1.49*∗*	12.27±1.54*∗*	38.16±1.44*∗*	10.53±1.28*∗*#
8 W	23.83±0.50	7.13±0.42	44.57±1.24*∗*	17.16±1.96*∗*	38.10±1.72*∗*#	12.82±1.64*∗*#	40.45±1.64*∗*	11.13±1.31#
10 W	24.55±0.40	7.20±0.50	46.38±1.85*∗*	19.02±2.00*∗*	39.73±1.95*∗*#	14.59±2.00*∗*	41.06±1.85*∗*#	13.88±1.60*∗*#

Note: NC: normal control mice, DN: diabetic nephropathy mice, DN+M: DN mice given 180 mg/kg metformin, and DN+AC: DN mice given 300 mg/kg AC. BW: body weight and FBG: basting blood glucose.  *∗*: *P*<0.05 compared with the NC group; #: *P*<0.05 compared with the DN group.

**Table 3:** Effect of AC on HbA1c and kidney/weight ratio in each group ($\overset{-}{x}$±SE).

Group	HbA1c (%)	K/W
NC	4.12±0.11	5.88±0.15
DN	7.61±0.31*∗*	7.47±0.70*∗*
DN+M	7.20±0.42*∗*	5.14±0.41#
DN+AC	6.53±0.41*∗*#	5.20±0.43#

Note: NC: normal control mice, DN: diabetic nephropathy mice, DN+M: DN mice given 180 mg/kg metformin, and DN+AC: DN mice given 300 mg/kg AC. K/W: Kidney/weight ratio. *∗*: *P*<0.05 compared with the NC group; #: *P*<0.05 compared with the DN group.

**Table 4:** Effect of AC on 24 h urine volume and 24 h urinary albumin excretion in each group ($\overset{-}{x}$±SE).

Weeks	NC		DN		DN+M		DN+AC	
	24 h UV (ml/24 h)	24 h UAE (*μ*g/24 h)	24 h UV (ml/24 h)	24 h UAE (*μ*g/24 h)	24 h UV (ml/24 h)	24 h UAE (*μ*g/24 h)	24 h UV (ml/24 h)	24 h UAE (*μ*g/24 h)
2 W	4.07±0.51	1.48±0.14	14.06±1.76*∗*	4.60±0.56*∗*	12.25±2.14*∗*	3.97±0.66*∗*	12.35±1.42*∗*	4.67±0.68*∗*
4 W	4.85±0.21	1.94±0.10	20.57±3.06*∗*	5.25±0.55*∗*	13.57±1.98*∗*#	4.49±0.58*∗*	15.64±2.03*∗*	4.57±0.36*∗*
6 W	5.14±0.30	1.54±0.11	23.50±3.18*∗*	5.80±0.81*∗*	18.35±2.17*∗*	4.61±0.37*∗*	17.56±1.17*∗*#	4.31±0.21*∗*#
8 W	4.93±0.43	1.34±0.07	26.00±2.92*∗*	6.39±0.63*∗*	18.28±2.27*∗*#	4.28±0.48*∗*#	17.06±1.50*∗*#	3.78±0.41*∗*#

Note: NC: normal control mice, DN: diabetic nephropathy mice, DN+M: DN mice given 180 mg/kg metformin, and DN+AC: DN mice given 300 mg/kg AC. 24 h UV: 24 h urine volume and 24 h UAE: 24 h urinary albumin excretion. *∗*: *P*<0.05 compared with the NC group; #: *P*<0.05 compared with the DN group.

## Data Availability

The data used to support the findings of this study are available from the corresponding author upon request.
